# Inspiratory and end-expiratory effects of lung recruitment in the prone position on dorsal lung aeration – new physiological insights in a secondary analysis of a randomised controlled study in post-cardiac surgery patients

**DOI:** 10.1016/j.bjao.2022.100105

**Published:** 2022-11-21

**Authors:** Andreas Martinsson, Erik Houltz, Andreas Wallinder, Jesper Magnusson, Sophie Lindgren, Ola Stenqvist, Anders Thorén

**Affiliations:** 1 Department of Anaesthesiology and Intensive Care Medicine, Sahlgrenska University Hospital, Gothenburg, Sweden; 2 Sahlgrenska Academy at University of Gothenburg, Gothenburg, Sweden; 3 Department of Cardiothoracic Surgery, Sahlgrenska University Hospital, Gothenburg, Sweden; 4 Department of Pulmonary Medicine, Sahlgrenska University Hospital, Gothenburg, Sweden

**Keywords:** atelectasis, cardiac surgery, electrical impedance tomography, end-expiratory lung volume, intra-tidal gas distribution, prone position, recruitment manoeuvre

## Abstract

**Background:**

Cardiac surgery produces dorso-basal atelectasis and ventilation/perfusion mismatch, associated with infection and prolonged intensive care. A postoperative lung volume recruitment manoeuvre to decrease the degree of atelectasis is routine. In patients with severe respiratory failure, prone positioning and recruitment manoeuvres may increase survival, oxygenation, or both. We compared the effects of lung recruitment in prone *vs* supine positions on dorsal inspiratory and end-expiratory lung aeration.

**Methods:**

In a prospective RCT, 30 post-cardiac surgery patients were randomly allocated to recruitment manoeuvres in the prone (*n*=15) or supine position (*n*=15). The primary endpoints were late dorsal inspiratory volume (arbitrary units [a.u.]) and left/right dorsal end-expiratory lung volume change (a.u.), prone *vs* supine after extubation, measured using electrical impedance tomography. Secondary outcomes included left/right dorsal inspiratory volumes (a.u.) and left/right dorsal end-expiratory lung volume change (a.u.) after prone recruitment and extubation.

**Results:**

The last part of dorsal end-inspiratory volume after extubation was higher after prone (49.1 a.u.; 95% confidence interval [CI], 37.4–60.6) *vs* supine recruitment (24.2 a.u.; 95% CI, 18.4–29.6; *P*=0.024). Improvement in left dorsal end-expiratory lung volume after extubation was higher after prone (382 a.u.; 95% CI, 261–502) *vs* supine recruitment (–71 a.u., 95% CI, –140 to –2; *n*=15; *P*<0.001). After prone recruitment, left *vs* right predominant end-expiratory dorsal lung volume change disappeared after extubation. However, both left and right end-expiratory volumes were higher in the prone group, after extubation.

**Conclusions:**

Recruitment in the prone position improves dorsal inspiratory and end-expiratory lung volumes after cardiac surgery.

**Clinical trial registration:**

NCT03009331.

Anaesthesia and controlled ventilation shift tidal ventilation from the dorsal to ventral lung regions.^1^ Cephalad displacement of the dorsal diaphragm and gravitational forces^2^ increase the dorsal pleural pressure,^3^ decreasing the end-expiratory lung volume and transpulmonary pressure, promoting atelectasis. Cardiac surgery patients may develop longstanding perioperative dorso-basal atelectasis^4^, ^5^, ^6^, ^7^, ^8^ with associated ventilation/perfusion mismatch.^9^^,^^10^ Postoperative recruitment manoeuvres^5^^,^^6^^,^^8^^,^^11^ improve oxygenation^12^ and reduce atelectasis, mechanically and inflammatory-induced lung injury,^9^^,^^13^, ^14^, ^15^ and risk of pneumonia,^9^^,^^16^ and prolonged ICU and hospital stay. A recent meta-analysis showed that recruitment manoeuvres after cardiac surgery may reduce postoperative pulmonary atelectasis, hypoxic events, and pneumonia also in patients with healthy lungs.^17^

The prone position is successfully used in acute respiratory disease syndrome (ARDS) to decrease mortality^18^ and in post-cardiac surgery patients to improve lung oxygenation^7^^,^^19^ and may be combined with recruitment manoeuvres.^20^^,^^21^ In the primary analysis of the present study, a short recruitment manoeuvre in the prone position was superior to a recruitment manoeuvre in the supine position in terms of dorsal lung aeration and oxygenation after extubation.^12^

As lung oxygenation mainly occurs during inspiration,^22^ it is important to optimise inspiratory dorsal aeration throughout inspiration after a recruitment manoeuvre. The inspiratory regional intra-tidal gas distribution^23^ adds information on inspiratory regional and temporal ventilation distribution and optimal PEEP setting.

The study aimed to investigate the differential effects of recruitment manoeuvres in the prone *vs* supine position on inspiratory and expiratory lung volumes measured by electrical impedance tomography (EIT).^24^ We hypothesised that recruitment manoeuvres in the prone position are superior, compared with supine recruitment, in improving dorsal inspiratory aeration and its timing and local end-expiratory lung volume after extubation, in post-cardiac surgery patients.

## Methods

### Study design

This is a secondary analysis of a prospective, randomised, controlled clinical trial in patients undergoing cardiac surgery,^12^ approved by the Gothenburg Ethics Committee (number: 371–17, 26 July 2017) and registered in ClinicalTrials.gov (NCT03009331) on 14 December 2016. Written informed consent was obtained from all subjects preoperatively, and enrolment was between August 2017 and March 2019. We followed the Consolidated Standards of Reporting Trials (CONSORT) recommendations on reporting randomised trials.

Adult patients (age >18 yr) undergoing on-pump cardiac surgery were included. The exclusion criteria were: (1) pulmonary disease, smoker or former smoker within 5 yr; (2) haemodynamic instability (norepinephrine infusion >0.20 μg kg min^−1^), milrinone infusion, or pacemaker dependency; (3) postoperative bleeding >100 ml h^−1^, or reoperation; (4) haemothorax or large pleural effusion detected using ultrasonography; (5) pneumothorax or air leak; and (6) postoperative PEEP >12 cm H_2_O or an inspired oxygen fraction (FiO_2_) >0.6.

### Anaesthesia and intraoperative management

See the primary analysis for a detailed description of anaesthesia and intraoperative management.^12^

### Study protocol

After arrival in the cardiothoracic intensive care unit (CTICU), the patients were prospectively randomly allocated (closed envelopes) to receive a recruitment manoeuvre in the supine or prone position (150–180°, left side up). At CTICU arrival, the patients were placed in a 20–30° head-up position with previously described ventilation (pressure-regulated volume-controlled mode, Servo-U; Maquet Critical Care, Solna, Sweden), and the ventilatory frequency adjusted to achieve normocapnia (*P*co_2_ 4.7–6.0 kPa). A propofol infusion (adjusted to achieve a Richmond Agitation–Sedation Scale score of –4) was used to prevent spontaneous breathing. A recruitment manoeuvre^25^ was performed in the supine (*n*=15) or prone position (*n*=15) after randomisation. PEEP was increased from 5 to 20 cm H_2_O in three steps over 30 s. The ventilator mode was switched to pressure control for the remaining part of the recruitment manoeuvre with an inspiratory pressure of 20 cm H_2_O above PEEP. The PEEP level was maintained at 20 cm H_2_O for 30 s, followed by decrements to 10 cm H_2_O in five steps over 2 min.

The experimental procedure, defining the S1–S4 time points, is illustrated in Supplementary Figure S1. The time from ICU admission to time point zero was 25–30 min. After time zero, there was a 25-min equilibration period before the first EIT data sampling period of 5 min (S1) in the supine position for both groups. In the prone group, the proning procedure plus recruitment manoeuvre (15 min) and de-proning (5 min) lasted 20 min. In the supine group, the recruitment manoeuvre was performed at the identical time point, preceded by a 10-min equilibration period. The second 5-min measurement period (S2) in the supine position for both groups, was performed after a new equilibration period of 5 and 10 min for the prone and supine groups, respectively. A further equilibration period started after S2 for 25 min, followed by a measurement period of 5 min (S3). After S3, the propofol was discontinued and after the patients emerged from sedation their tracheas were extubated. After extubation, there was a 25-min equilibration period followed by S4. For all patients, data are from the supine position (S1–S4). Thus, the difference in intervention between the groups was that the recruitment manoeuvre of 5 min took place in the prone position in the prone group, who were also maintained in the prone position for 20 min.

### Electrical impedance tomography

For description of the EIT method and data sampling, see the primary analysis.^12^

EIT images are based on bioimpedance variation (ΔZ), with multiple display options. Based on the reference section's tidal image, the ventilated area is divided into two equally large surfaces, a ventral and a dorsal region. The dorsal region was further divided into equally large left and right regions. Intra-tidal gas distribution is a non-cumulative, temporal impedance variation display, dividing inspiration into six iso-volumetric partitions,^23^ parts 1–6, where end-inspiratory aeration is referred to as part 6. The dorsal *vs* ventral portion is expressed as a percentage or arbitrary volume. The six iso-volumetric parts may be presented as cumulative volume, where the sum of the six volumes represents the tidal ventilation in the area of interest.

In the baseline measurement period (S1), tidal ΔZ was calibrated to the V_T_, enabling the calculation of inspiratory lung volumes and end-expiratory lung volume change (ΔEELV) in the subsequent measurement periods (S2–S4).

### Co-primary outcomes

The primary outcome measures were late dorsal inspiratory volume (a.u.), and left and right dorsal ΔEELV (a.u.) after extubation (S4), in patients receiving a prone *vs* supine recruitment manoeuvre.

### Secondary outcomes

Secondary outcome measures were left and right dorsal inspiratory volume (a.u.) after the recruitment manoeuvre (S3) and after extubation (S4), in patients receiving a prone *vs* supine recruitment manoeuvre. Furthermore, we compared left *vs* right dorsal ΔEELV (a.u.) after a prone recruitment manoeuvre, before (S3) and after extubation (S4).

### Statistical analysis

**Fig 1:**
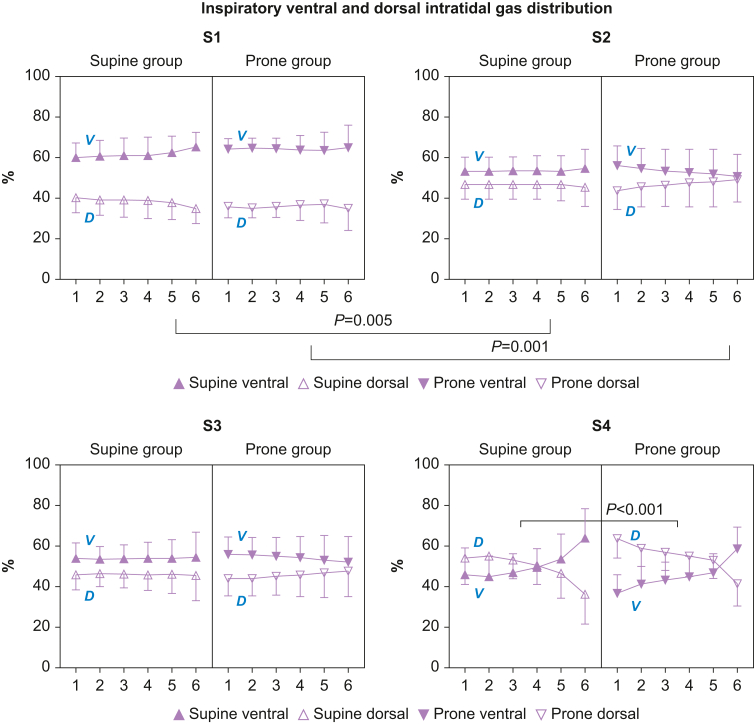
Intra-tidal gas distribution (ITV) in six iso-volumetric partitions, 1–6, represented as a percentage of inspiratory tidal volume divided in one ventral and one dorsal region. Ventral inspiratory predominance in S1 is attenuated in both groups after recruitment manoeuvre in S2 and S3, suggesting ventilatory homogeneity. After extubation (S4), dorsal inspiratory gas distribution predominance was protracted in the prone *vs* the supine group. S1, before the recruitment manoeuvre; S2, immediately after the recruitment manoeuvre; S3, 30 min after the recruitment manoeuvre; S4, 30 min after extubation during spontaneous ventilation. %, percentage of inspiratory tidal volume; D, dorsal region; V, ventral region.

**Fig 2:**
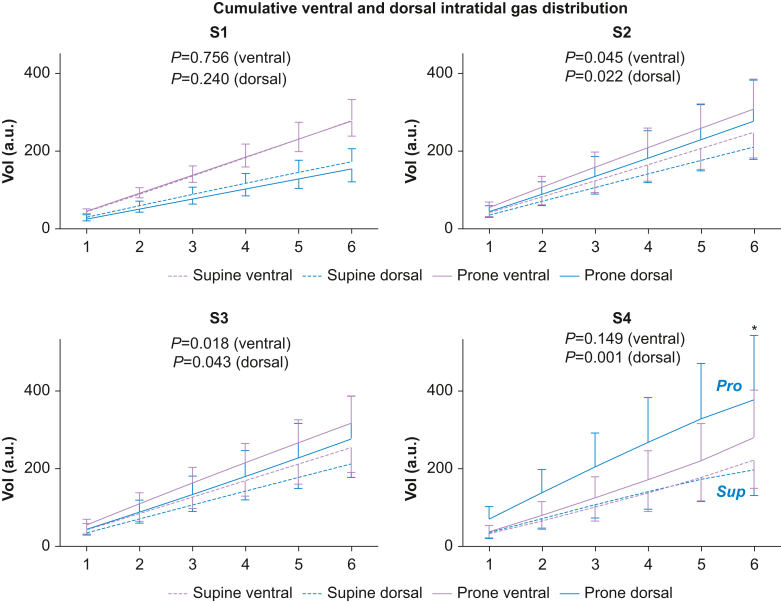
Intra-tidal gas distribution (ITV) in six iso-volumetric partitions, 1–6, represented as cumulative inspiratory tidal volume divided into one ventral and one dorsal region. After extubation (S4), cumulative and end-inspiratory (part 6) volumes are larger in the prone *vs* the supine group. S1, before the recruitment manoeuvre; S2, immediately after the recruitment manoeuvre; S3, 30 min after the recruitment manoeuvre; S4, 30 min after extubation during spontaneous ventilation. a.u., arbitrary unit; Pro, prone group; Sup, supine group; Vol, volume.

**Fig 3:**
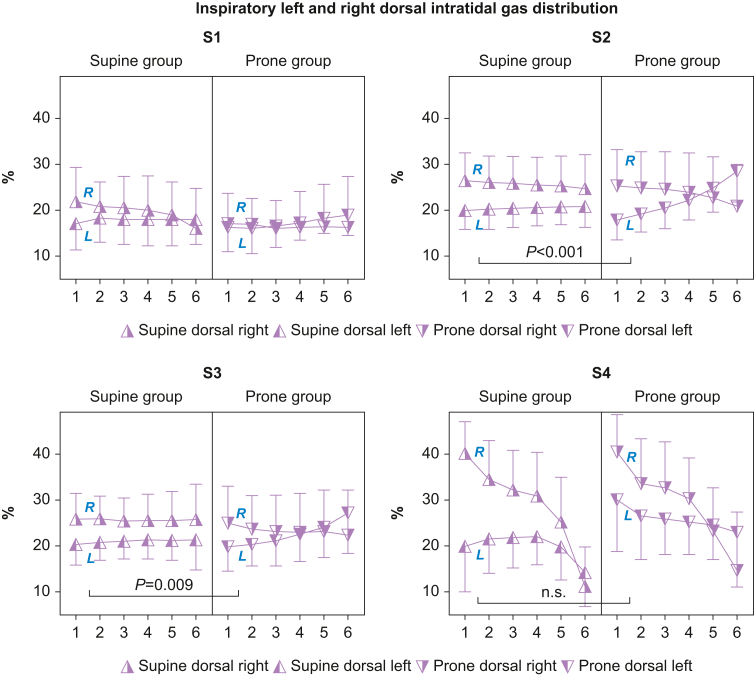
Dorsal intra-tidal gas distribution (ITV) in six iso-volumetric partitions, 1–6, represented as a percentage of inspiratory tidal volume divided on left and right sides. After the recruitment manoeuvre in S2 and S3, left dorsal inspiratory gas distribution is larger in the prone *vs* the supine group. After extubation in S4, the prone group left dorsal side advantage is lost, suggesting a regional proportionate de-recruitment. There is a right dorsal side predominance in both groups after extubation. S1, before the recruitment manoeuvre; S2, immediately after the recruitment manoeuvre; S3, 30 min after the recruitment manoeuvre; S4, 30 min after extubation during spontaneous ventilation. %, percentage of inspiratory tidal volume; L, left side; R, right side.

**Fig 4:**
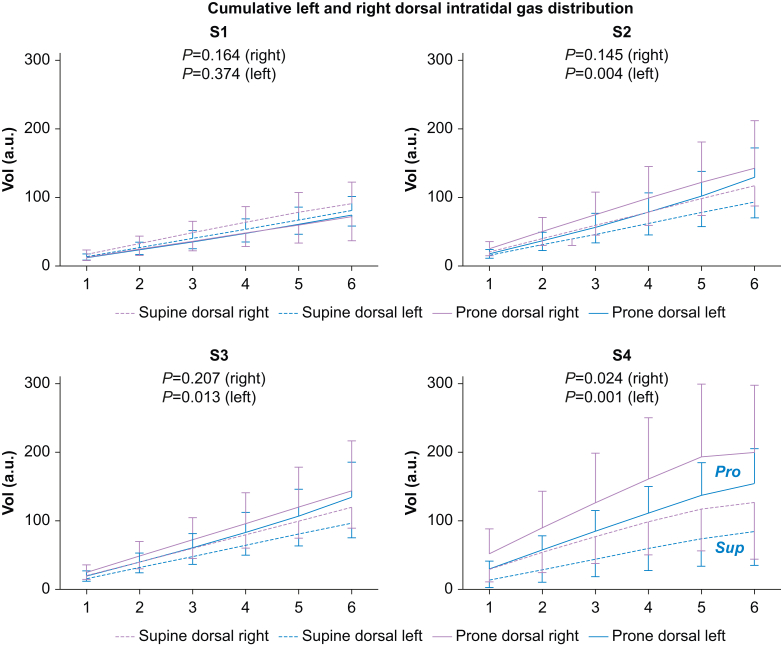
Dorsal intra-tidal gas distribution (ITV) in six iso-volumetric partitions, 1–6, represented as cumulative inspiratory tidal volume divided on left and right sides. After the recruitment manoeuvre in S2 and S3, left side cumulative inspiratory volumes are larger in the prone *vs* the supine group. After extubation in S4, left and right side cumulative inspiratory volumes are larger in the prone *vs* the supine group. S1, before the recruitment manoeuvre; S2, immediately after the recruitment manoeuvre; S3, 30 min after the recruitment manoeuvre; S4, 30 min after extubation during spontaneous ventilation. a.u., arbitrary unit; Pro, Prone group; Sup, Supine group; Vol, volume.

**Fig 5:**
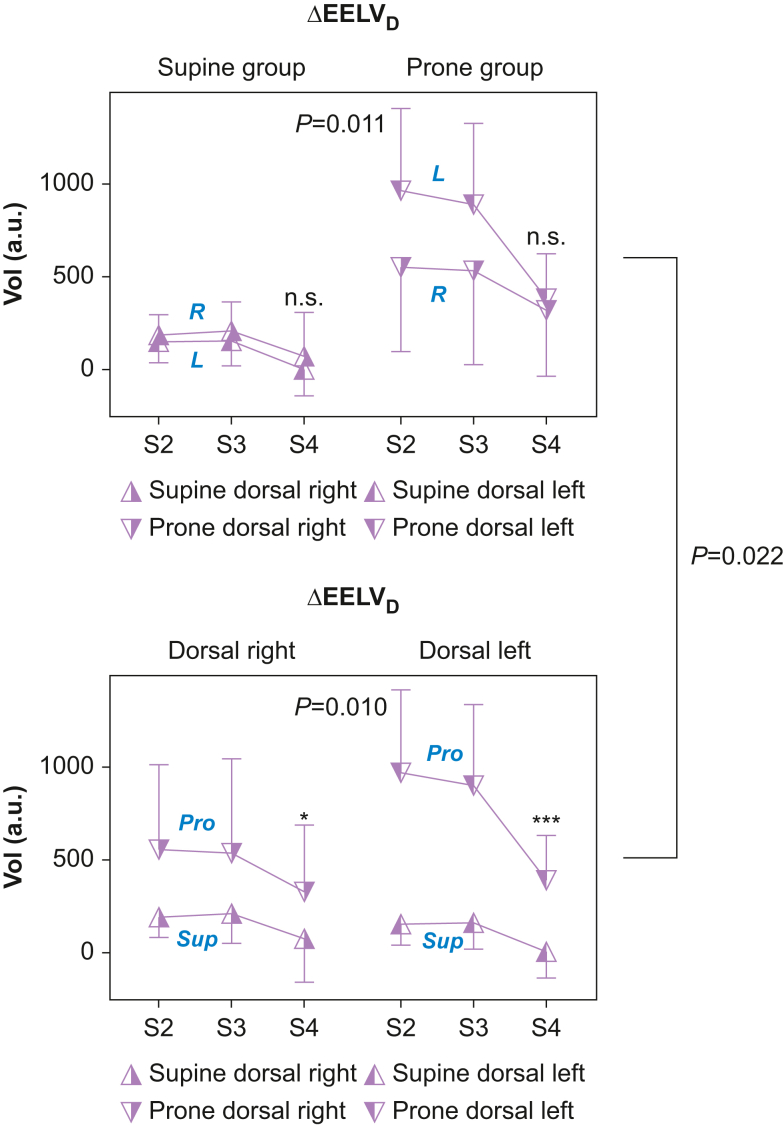
Dorsal delta end-expiratory lung volume (ΔEELV_D_). Upper: supine and prone groups, ΔEELV_D_ divided into left and right sides. Lower: left and right side, respectively, comparison between supine and prone group ΔEELV_D_. Left and right ΔEELV_D_ are larger in the prone *vs* supine group after the recruitment manoeuvre and after extubation. In the prone group, left *vs* right ΔEELV_D_ is larger after the recruitment manoeuvre, but this superiority is lost after extubation. S2, immediately after recruitment manoeuvre; S3, 30 min after the recruitment manoeuvre; S4, 30 min after extubation during spontaneous ventilation. a.u., arbitrary unit; Pro, Prone group; Sup, Supine group; Vol, volume.

The Shapiro–Wilks test confirmed normal distribution. Analyses were performed using SPSS ver. 24 (IBM Corp., Chicago, IL, USA). We did not perform *post-hoc* power analysis as it is not recommended.^26^ Two-way analysis of variance (anova) for repeated measurements (time *vs* group) was used to evaluate differences between the groups (Fig 1, Fig 2, Fig 3, Fig 4, Fig 5; Table 1, Table 2; and Supplementary Tables S1 and S2). Three-way anova for repeated measurements, with position and side as independent variables, was used to detect a difference in dorsal ΔEELV (Fig. 5). A two-way anova sub-analysis demonstrated the effect of side and position over time (Fig. 5, Table 2). For other analyses, in Figure 2 (S4), Figure 5 (S4), and Table 1, Table 2 (S4), a *t*-test was used.

**Table 1:** Calculated respiratory data – regional cumulative tidal volumes and ITV partition no. 6 (a.u.). Calculated ventral and dorsal cumulative regional tidal volumes (upper section), and partition no. 6 tidal volumes (lower section), S1–S4, in supine position. Data are presented as mean (standard deviation). anova, analysis of variance; a.u.: arbitrary unit; EIT, electrical impedance tomography; ITV 6, intra-tidal gas distribution partition no. 6 of 6. anova represents full inspiratory comparison supine to prone (inspiratory partition nos. 1–6), *t*-test represents end-inspiratory comparison supine with prone (inspiratory partition no. 6).

Statistical method		S1		S2		S3		S4		
			Anova		Anova		Anova		Anova	*t*-Test
*EIT variables*										
ITV ventral (a.u.)	Supine	280.1 (52.8)	0.756	247.8 (65.9)	0.045	254.4 (64.0)	0.018	223.5 (72.8)	0.149	
	Prone	277.5 (38.4)		307.1 (77.9)		317.3 (69.5)		282.4 (120.4)		
ITV dorsal (a.u.)	Supine	172.6 (33.7)	0.240	209.1 (30.9)	0.022	212.4 (34.9)	0.043	197.4 (64.45)	0.001	
	Prone	154.9 (33.3)		276.6 (95.9)		276.8 (110.2)		378.2 (164.9)		
ITV 6 ventral (a.u.)	Supine	49.5 (9.7)		42.4 (12.8)		43.0 (13.8)		45.9 (15.2)		
	Prone	46.7 (8.8)		49.4 (15.4)		50.4 (13.1)		61.1 (15.7)		
ITV 6 ventral (a.u.)	Supine	25.9 (4.7)		33.7 (5.0)		34.9 (8.6)		24.2 (11.3)		0.024
	Prone	25.4 (9.5)		47.9 (15.8)		48.6 (22.8)		49.1 (13.1)

**Table 2:** Calculated respiratory data – ΔEELV (a.u.). Calculated dorsal left and dorsal right ΔEELV, data registered in supine position, S2–S4. Data are presented as mean (standard deviation). ΔEELV, delta end-expiratory lung volume; anova, analysis of variance; a.u., arbitrary unit; EIT, electrical impedance tomography.

Statistical method		S2	S3	S4		
					*t*-Test	Anova
*EIT variables*						
Dorsal left ΔEELV (a.u.)	Supine	151.3 (117.9)	157.4 (136.6)	–70.7 (137.8)	<0.001	<0.001
	Prone	954.1 (443.2)	888.0 (492.7)	382.0 (239.8)		
Dorsal right ΔEELV (a.u.)	Supine	189.2 (107.0)	204.4 (158.0)	–13.5 (234.0)	0.041	0.005
	Prone	549.8 (451.7)	529.6 (501)	321.2 (353.2)		

A *P*-value <0.05 was considered statistically significant. Results are presented as mean with standard deviation (sd). In the figures, ∗*P*<0.05, ∗∗*P*<0.01.

## Results

For patient and perioperative data, ventilatory settings and the CONSORT diagram, see the primary study.^12^

### Inspiratory ventral and dorsal intra-tidal gas distribution (%)

There was a significantly increased ventilatory homogeneity after recruitment (S2) in both groups (Fig. 1 and Supplementary Table S1). After extubation (S4) there was a significantly higher dorsal aeration throughout inspiration in the prone group compared with the supine group, and a later inspiratory shift from dorsal to ventral gas distribution.

### Cumulative ventral and dorsal intra-tidal gas distribution (a.u.)

The co-primary endpoint: end-inspiratory dorsal volume part 6 after extubation (S4) was higher after prone (49.1 a.u.; 95% confidence interval [CI], 37.4–60.6) *vs* supine recruitment (24.2 a.u.; 95% CI, 18.4–29.6; *P*=0.024) (Fig. 2, Table 1, and Supplementary Table S3). The *mean difference* of end-inspiratory dorsal volume part 6 after extubation, prone *vs* supine, was 24.8 a.u. (95% CI, 11.3–38.3). This mean difference is considered clinically/physiologically significant.

The cumulative dorsal inspiratory volumes were significantly higher after recruitment (S2, S3) and after extubation (S4) in the prone *vs* supine group*.*

### Inspiratory left and right dorsal intra-tidal gas distribution (%)

The significantly superior end-inspiratory dorsal aeration in left *vs* right dorsal lung regions after prone *vs* supine recruitment (S2, S3) disappeared after extubation (S4) (Fig. 3 and Supplementary Table S1).

### Cumulative left and right dorsal intra-tidal gas distribution (a.u.)

After extubation (S4), the cumulative dorsal inspiratory volumes in both left and right dorsal region were significantly higher in the prone *vs* supine group (Fig. 4 and Supplementary Table S2).

### Dorsal change in left and right end-expiratory lung volume (ΔEELV_D_ [a.u.])

Interaction analyses showed both position and side dependent effects (*P*=0.022). The co-primary endpoint left dorsal ΔEELV after extubation (S4, Fig. 5, lower panel) was higher in the prone group (382 a.u.; 95% CI, 261–502) *vs* the supine group (–71 a.u.; 95% CI, –140 to –2; *P*<0.001) (Fig. 5, Table 2, and Supplementary Table S3). The *last co-primary endpoint* right dorsal ΔEELV after extubation (S4, Fig. 5, lower panel) was higher in the prone group (321 a.u.; 95% CI, 142–499) *vs* the supine group (–14 a.u.; 95% CI, –131 to 105; *P*=0.041). The *mean difference* of left dorsal ΔEELV after extubation, prone *vs* supine, was 452 a.u. (95% CI, 306–599). The *mean difference* of right dorsal ΔEELV after extubation, prone *vs* supine, was 335 a.u. (95% CI, 111–559). These mean differences are considered clinically/physiologically significant.

The secondary endpoint left *vs* right dorsal ΔEELV (Fig. 5, upper panel) after prone recruitment (S2) was 954 a.u. (95% CI, 730–1177) *vs* 549 a.u. (95% CI, 320–777). This difference disappeared after extubation.

## Discussion

The current randomised controlled study is the first to use intra-tidal gas distribution analyses to demonstrate that a recruitment manoeuvre in the prone position causes a more pronounced increase in inspiratory aeration when compared with a recruitment manoeuvre in the supine position. Furthermore, when lung aeration was measured after extubation, recruitment in the prone position was superior regarding dorsal inspiratory aeration and end-expiratory lung volume of both left (LLL) and right lower lobes (RLL). The superior end-expiratory aeration after prone recruitment in the left *vs* right lower lung lobe disappeared after extubation.

The validation and accuracy of EIT after cardiac surgery and in ARDS patients to detect changes in regional lung volumes were previously described in the primary analysis. The safety aspects of using the prone position in post-cardiac surgical patients are under debate but in a recent review,^27^ no complications were seen in the majority of the 230 adult cardiac surgery patients studied: only minor complications were described in the earlier studies. With skilled staff experienced in positioning COVID patients in the prone position, together with using only a short period of 20 min in the prone position, as in the present study, the risk of complications can be minimised.

Using intra-tidal gas distribution in a decremental PEEP trial in post-cardiac surgery patients, the optimal PEEP level was 10–12 cm of H_2_O, expressed as the most inspiratory homogenous ventral/dorsal aeration.^6^ In the present study, a PEEP of 10 cm of H_2_O was used after the recruitment manoeuvres, which improved homogeneity through an increase in inspiratory dorsal aeration in both groups. Interestingly, there was a superior inspiratory dorsal intra-tidal aeration in the prone *vs* supine group (equilibration period) after extubation. This difference was sustained throughout the inspiration, including the late dorsal inspiratory volume.

In a previous study on post-cardiac surgery patients, using the intra-tidal gas distribution analysis, pressure support ventilation, compared with pressure-controlled ventilation, improved dorsal ventilation by increasing dorsal diaphragm contraction, particularly during lower tidal volumes (V_T_). However, this superior dorsal aeration during pressure support ventilation lasted only during half of the inspiration, probably because of a late inspiratory de-recruitment of dorsal areas caused by too high V_T_, pressure support, or both, which in turn, decrease the dorsal diaphragm contraction. Furthermore, the selected PEEP level in that study was not preceded by a recruitment manoeuvre, which may contribute to the late inspiratory dorsal de-recruitment.^28^ In addition, the findings of that study emphasise the importance of the use of assisted ventilation, or even better, early extubation, to activate the diaphragm. In general, extubation plus the sitting position improves dorsal V_T_, end-expiratory lung volume (EELV), ventilation/perfusion matching and functional residual capacity, and a decrease in respiratory system resistance and abdominal pressure.^12^^,^^29^^,^^30^

In the present study, after extubation and during spontaneous ventilation, we confirmed (Fig. 1) the previously described ventral/dorsal intra-tidal gas distribution analysis pattern shown in spontaneously breathing healthy patients. A predominant dorsal inspiratory aeration caused by dorsal diaphragmatic contraction is seen during the first 50% of the inspiration, whereas anterior diaphragmic and thoracic cage muscles complete the later inspiration phase, leading to a dominating ventral ventilation at the end of inspiration.^31^ Interestingly, in the extubated prone group compared with the supine group, there was a longer and higher inspiratory domination of dorsal aeration, most likely caused by a more efficient and extended dorsal diaphragm contraction.^32^

In patients undergoing spinal surgery, without lung recruitment, the intra-tidal gas distribution pattern in the supine and prone positions was compared at different PEEP levels. Remarkably, turning the patient prone *per se* altered the inspiratory regional ventilation from a ventral to dorsal domination. However, despite using the prone position and a PEEP of 12 cm H_2_O, a lack of ventral/dorsal homogeneity was observed.^33^ In contrast, in the present study, the lung-recruited patients in both groups showed a better homogeneity, reflecting the importance of recruitment manoeuvres in cardiac surgical with healthy lungs or orthopaedic patients,^33^ as ventilatory homogeneity reduces local lung stress and strain.^34^

In spontaneously breathing awake patients undergoing prostatectomy,^35^ in the sitting or supine position, the shift to mechanical ventilation was assessed using the EIT-derived centre of ventilation variable.^31^ During spontaneous ventilation, less dorsal atelectasis and improved dorsal ventilation were seen in the sitting *vs* supine position. When switching to mechanical ventilation in the sitting position, ventral redistribution and dorsal silent spaces occurred, indicating atelectasis formation in dorsal areas.^35^ In the present study, the sitting position combined with extubation caused a dorso-basal alveolar recruitment, shown by a dorsal increase in inspiratory volume, which was more pronounced and better maintained in the prone group. Furthermore, in a previous study in post-cardiac surgical mechanically ventilated patients, the semi-recumbent position of 30° improved EELV and dorsal aeration,^30^ but without improvement in lung oxygenation. In contrast, we showed in the primary analysis that *extubated* prone-recruited patients in the sitting position after cardiac surgery improved their lung oxygenation by use of their dorsal diaphragm.^1^ With the new application of the intra-tidal gas distribution analysis, late inspiratory de-recruitment (Fig 1, Fig 2) after extubation can be identified and reduced by prone recruitment, which promotes a longer inspiratory oxygen uptake and lung oxygenation.^22^ ^36^ This may partly explain the superior oxygenation in the prone group seen in the primary study.

What then are the mechanisms behind the superior recruitment of the dorsal regions when performed in the prone position? With healthy lungs, the prone position increases EELV, transpulmonary end-inspiratory and end-expiratory pressure, but without a change in transpulmonary driving pressure.^29^^,^^37^ The superior dorsal recruitment achieved by using the prone position in the present study could be explained by an increase in anterior chest wall elastance with the lungs operating between two rigid bars, the sternum and the spine,^29^^,^^37^, ^38^, ^39^ promoting the distribution of inspired air to the dorsal regions. This, together with the unloading of the pressure exerted by the heart and mediastinum^34^^,^^40^ particularly on the LLL, would promote dorsal recruitment. In other words, as the sternum cannot move downwards in the prone position and the spine is stiff by nature, together with gravitational forces on the heart, mediastinum, and perialveolar vascular bed, the increase in lung volumes during prone recruitment endorses *dorsal* alveolar recruitment. These positive effects remained after patients were turned supine.

The development of dorso-basal atelectasis after cardiac surgery is more pronounced in the LLL^30^^,^^41^ compared with the RLL and has been suggested to be caused by apnoea during CPB, gravitational forces from the heart and mediastinum, retraction of the LLL, pleural trauma, preparation of the left internal mammary artery, transient paresis of the dorsal left phrenic nerve, and increased bypass time.^42^ In the present study, the superior dorsal ΔEELV in the LLL compared with RLL lobe after a prone recruitment manoeuvre disappeared after extubation. It is most likely explained by a cessation of positive pressure ventilation and the gravitational forces from the heart and mediastinum on the LLL.^40^ The new information on changes in left- and right-sided end-expiratory lung volumes indicate therapeutic options to increase local aeration.

A limitation of the present study is that we did not measure respiratory mechanics during and after the recruitment manoeuvres. However, the primary endpoints were differences in inspiratory and end-expiratory temporal and regional aeration after extubation. A second limitation is that we did not study long-term effects and aeration beyond 30 min after extubation, but interestingly, in the primary analysis patients undergoing the recruitment manoeuvre in the prone position required less supplemental oxygen until the second postoperative day. Earlier studies, using the postoperative open-lung concept, increased functional residual capacity for up to 5 days postoperatively.^13^^,^^14^ Furthermore, a recent meta-analysis showed that recruitment manoeuvres after cardiac surgery may reduce postoperative pulmonary atelectasis, hypoxic events, and pneumonia in patients with healthy lungs.^17^

## Conclusions

Our new application of inspiratory regional intra-tidal gas distribution analysis, showed that a lung recruitment manoeuvre in the prone *vs* supine position is superior regarding dorsal inspiratory aeration. In addition, dorsal end-expiratory aeration was improved in both lower lobes. Although an early postoperative prone recruitment manoeuvre after cardiac surgery is safe in experienced hands, future studies are necessary to evaluate potential long-term beneficial effects on lung oxygenation before this method can be generally recommended. Studies to evaluate the prone recruitment method in patients with reduced left ventricular ejection fraction, obesity, or both, who are at greater risk of developing postoperative pulmonary complications, might justify the use in these groups.^16^^,^^43^^,^^44^

## Funding

Sahlgrenska University Hospital (ALFGBG-867311).

## Authors' contributions

Conduct of experiments: AM

Data analysis: AM

Statistical analysis: AM, EH

Data interpretation: AT, AM, SL, AW, OS, JM

Wrote the first draft of the manuscript: AM

Manuscript revision: EH, AW, SL, JM, OS, AT

All authors gave final approval of the submitted version.
