# Supplementary material for: Inspiratory and end-expiratory effects of lung recruitment in the prone position on dorsal lung aeration – new physiological insights in a secondary analysis of a randomised controlled study in post-cardiac surgery patients
Source: BJA Open. 2022 Nov 21;4:100105. doi: 10.1016/j.bjao.2022.100105 (PMC10430825; doi:10.1016/j.bjao.2022.100105)
Supplement: Multimedia component 1 [file mmc1.docx]

**
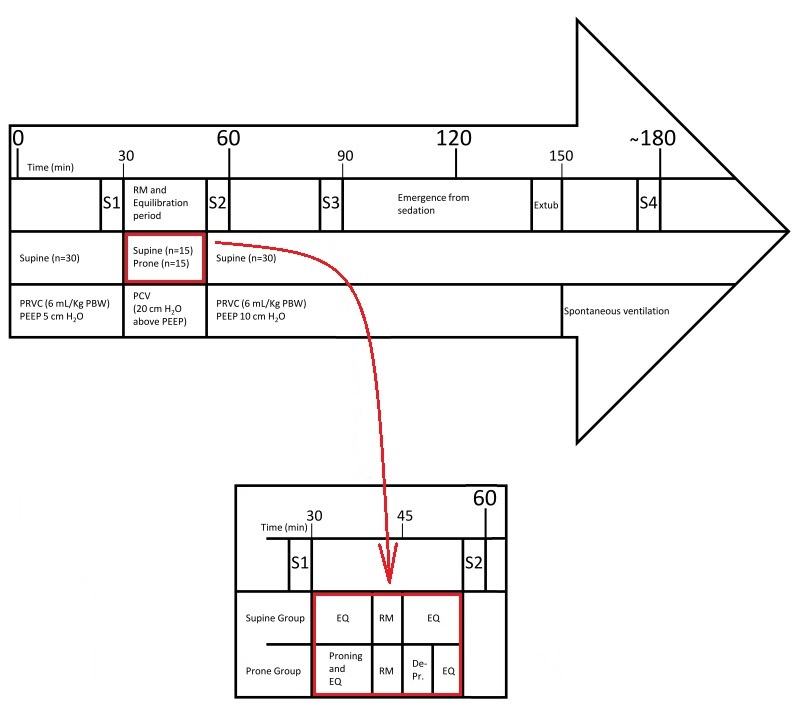
Figure SuF1: Timeline**

Figure SuF1: Schematic drawing of the experimental procedure. S1-S4: Supine position registration and measuring points; RM: Recruitment Manoeuvre; Extub: Extubation; PRVC: Pressure Regulated Volume Control; PCV: Pressure Control Ventilation; PEEP: Positive End-Expiratory Pressure; EQ: Equilibration Period; De-Pr.: De-proning

Table Su1. Collected respiratory data, registered in supine position, S1-S4. Upper section: Ventral and dorsal regional partition no. 6 tidal variation (%). Lower section: Dorsal left and dorsal right regional partition no. 6 tidal variation (%). Data are presented as mean (standard deviation). ITV 6; Intratidal gas distribution partition no. 6 of 6; ANOVA represents full inspiratory comparison supine to prone (inspiratory partition no. 1-6).

Table Su2. Calculated dorsal left and dorsal right cumulative regional tidal volumes, S1-S4, in supine position. Data are presented as mean (standard deviation). ITV; Intratidal gas distribution; a.u.: Arbitrary unit; ANOVA represents full inspiratory comparison supine to prone (inspiratory partition no. 1-6).

Table Su3. Mean difference of calculated dorsal partition no. 6 tidal volumes (data registered in supine position, S1-S4), and mean difference of calculated dorsal left and dorsal right ∆EELV (data registered in supine position, S2-S4). Data are presented as mean (standard deviation). ITV: Intratidal gas distribution; a.u.: Arbitrary unit; ∆EELV: Delta End-Expiratory Lung Volume; Ci: Confidence interval.
